# Diphtheria

**Published:** 1884-02-20

**Authors:** R. S. Powell

**Affiliations:** Virginia


### DIPHTHERIA.

BY R. S. POWELL, M.D., OF VA.

I have just received a report of the proceedings of the Virginia Medical Society, in which I see the discussion of Diphtheria prominently engaged the attention of that body. I have, also, just learned that a prominent citizen of Petersburg has just died with the same malady, notwithstanding he was treated by my distinguished friend and fellow-countryman, John Herbert Claiborne, a man of whom every true physician is prond, and whom every true Virginian delights to honor; notwithstanding this fact, death did its work.

Before proceeding to discuss the treatment of this disease, I would say that I have been practicing medicine more than twenty-six years, and have no disposition to write for the sake of notoriety. The apology I offer for troubling you with this communication is alone in the interest of humanity.

The first time my attention was called to this disease was in 1853 or 1854. Mr. Ben King had an interesting family of children, two of whom were stricken down with the disease in a very malignant form, and—notwithstanding they were treated by the best medical men in this section of the country—they both died, and the father soon followed the children to the grave with the same disease. The disease was supposed to be caused by a damp cellar under the house. So thoroughly convinced were the medical men of this fact that Mr. King ordered the house to be moved, which was done after his death. The treatment, as well as I remember, consisted in touching the diseased throat with caustic and giving a little constitutional treatment.

In 1858 I had located in the Southern portion of this county, and this same disease broke out in my immediate vicinity, not slighting the mansions of the rich nor the hovels of the poor. Remembering the treatment that had resulted in death in the King family, I determined to make a new departure. I dissolved a teaspoonful of pulverized copperas with a tablespoonful of common salt in about half a pint of water. After mixing well I wrapped a thin piece of cotton or linen rag around my finger, and after wetting it in this solution I inserted it into the patient's throat and completely wiped off every particle of epithelial matter. I threw that rag into the fire and repeated the operation with a clean rag so as to get the solution in contact with the diseased surface. This destroys the fungus growth and prevents a reformation of the same. I require the patient to use a gargle of salt and alum as often as the severity of the case may indicate, and, also, to counter-irritate the throat with a flannel rag wrung out of hot salt and water, applied as warm as the patient can bear it. If the patient is much debilitated or nervous, I give small quantities of brandy and quinine at short intervals. If this treatment is persisted in, it will cure a majority of cases in from three to six days. I remember being called to see a very bad little boy about eight years of age. He was sitting in his mother's lap, breathing badly, with a bloody discharge from his nose. I had to prize his teeth apart, so as to insert my finger and, as it were, to scour out the filth that was blockading the pathway to life. When I returned the next day he was well enough to be at the door. As soon as he saw me he ran under the bed, and as I pulled him out bv the foot he cried out, "Let me alone. I am asleep." He was well in a few days, and is now a prosperous farmer.

I think I must have seen more than one hundred cases during this epidemic, and not a single fatal case occurred in my practice that I saw within forty-eight hours after the inception of the disease.

I would no more hesitate to use force to wash out this death-dealing matter and save human life, than I would to use the power of anæsthesia to control the muscular action of a strong man who had his leg crushed in order to amputate it and save his life.

This, to some, may look rash. But what is it that our profession is not warranted in doing to save life? I would state that while this epidemic was raging Dr. H., an old and eminent physician, stuck to the "let alone theory," and lost every case. About his last case was that of his little niece, a sprightly little girl about twelve years of age. When he saw that his treatment was futile he sent for me. When I arrived he confessed that he knew nothing about the disease, and begged that I would do all I could to save her life. I soon returned and told him the child was then dying, the disease having extended into the trachea. I invariably told my patrons if they did not send for me within forty-eight hours after the patient was taken, not to send at all. The main thing in the treatment is to meet the disease at the outset and combat it from its very inception, before it has poisoned the system and complicated the case.

In conclusion I would say that if this treatment does not cure, the disease has changed its character, or the remedies have lost their virtue.

